# Cemented or cementless total knee arthroplasty? Comparative results of 200 cases at a minimum follow-up of 11 years

**DOI:** 10.1051/sicotj/2017046

**Published:** 2017-12-12

**Authors:** Jean-Louis Prudhon, Régis Verdier

**Affiliations:** 1 Centre ostéo-articulaire 5 rue Raoul Blanchard 38000 Grenoble France; 2 Groupe Lépine 175 rue Jacquard CS 50307 69727 Genay Cedex France

**Keywords:** Total knee arthroplasty, Cemented, Cementless, Comparative series, Hydroxyapatite

## Abstract

*Introduction:* Since 1996 we have been using cementless fixation with hydroxyapatite (HA) coating. The purpose of this paper is to compare survivorship of a series of 100 cemented Total Knee Arthroplasty (TKA) to a similar series of 100 cementless with a follow up of 11–16 years.

*Material methods*: Both TKA are mobile bearing total knee postero-stabilized. They can be used with cement or without cement. Among 1030 New Wave TKATM implanted from 2002 to 2015 we have identified 100 cemented TKAs and 100 cementless TKAs. All these cases were primary replacement. Differences in survival probability were determined using log-rank test.

*Results*: Survival probabilities at 11 years of follow-up were: Cemented group: 90.2% CI95% [81.9–94.8]; Cementless group: 95.4% CI95% [88.1–98.2]. Comparison between both group showed significant difference, *p* = 0.32.

*Discussion*: The advantages of cementless TKA are bone stock preservation, cement debris protection and the potential to achieve biologic fixation. Cementless implants rely on a porous or roughened surface to facilitate bone formation. HA has been shown to accelerate bone integration and to decrease micro motion of the components and to increase fixation. With a survival probability of 90.2% (cemented version) and 95.4% (cementless version), this total knee prosthesis performs as intended in primary total knee arthroplasty. No statistical differences could be found between cemented and cementless implants.

## Introduction – background

Total knee arthroplasty (TKA) is a reliable procedure to treat severe cartilage disease of the knee. Primary osteo-arthritis (OA) is the main cause but cartilage destruction may occur after a traumatic event such as a ligament injury or an articular fracture. The frontal deviation of the lower limb is the main risk factor for degenerative changes. Obesity, gender, activity level and age are also well identified as risk factors. The destruction resulting from rheumatoid arthritis is rare but it affects a young population who have severe multiple joint involvement. The TKA outcomes are generally good with a mean survivorship of 90% at 10 years [1, 2]. Over three decades improvements have been proposed concerning implant design, surgical techniques, as well as pain management and evaluation of outcome. Regarding implant fixation, the most common method to secure immediate and long-term fixation is dedicated to a cementing technique. In 1980 the first generation of cementless implants [PCA (porous coated Howmedica)] was introduced. Hungerford and co-workers [3–5] published the first clinical results with mid-term results similar to those of the cemented prostheses. Even though the fixation process was much different to the contemporary design, most of the TKA implants over the world are currently cemented.

Since 1996 we have been using cementless fixation with hydroxyapatite (HA) coating as proposed by Epinette and Manley [6].

The purpose of this paper is to compare a series of 100 cemented New Wave TKA^TM^ (groupe lépine, Genay, France) to a similar series of 100 cementless New Wave TKA^TM^ with a follow-up of 11–16 years and:

1. to analyse complications and reasons for revision (major or minor revision),
2. to report on survival rate at 10 years.

## Materials and methods

### Implant characteristics

New Wave TKA^TM^ is a mobile bearing total knee postero-stabilized.

**Figure 1.**
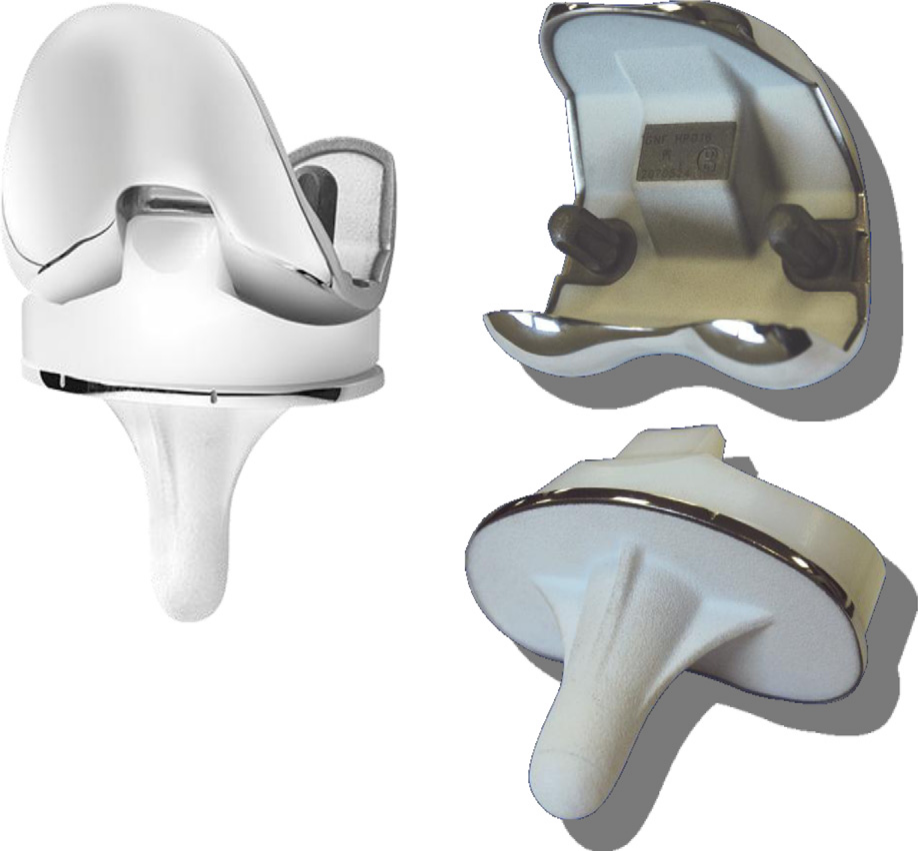
New Wave TKA^TM^ implant design, HA coating in cementless version.

**Figure 2.**
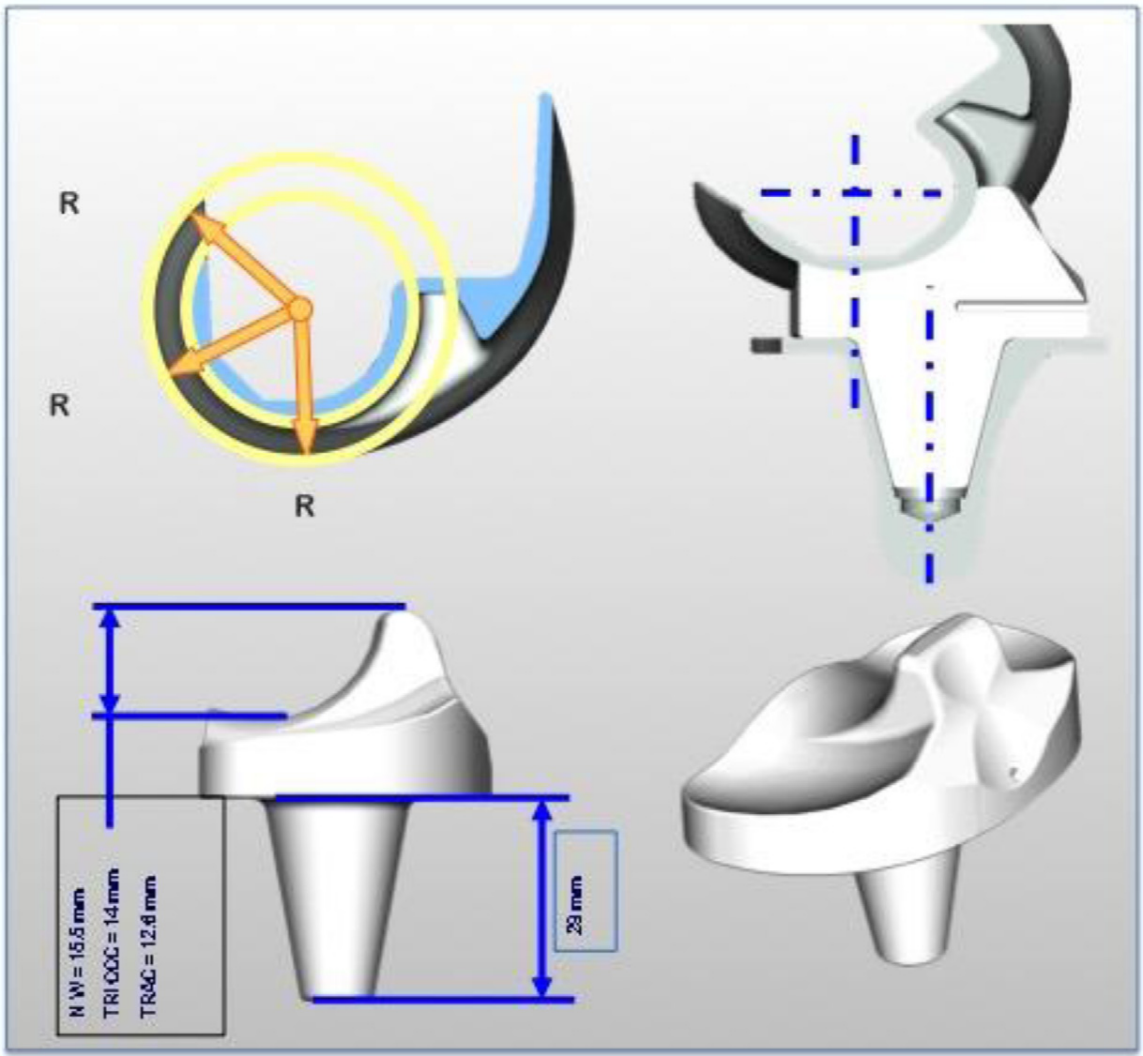
New Wave TKA^TM^ implant features.

The femoral condyle is symmetric, made of cobalt chromium alloy. It can be used with or without cement. In the cementless version, the inner implant is completely coated under vacuum with a double layer of titanium spray (120 μm) covered with HA (80 μm) (Figure 1). The condyle has one single rotation centre (Figure 2).

The tibia plate has been designed to get the maximum contact between the host bone and the implant. The stem is perpendicular to the tibia plate in the frontal and sagittal plane. Two types of cementless tibial trays can be used:

1. a regular stem 40 mm long fully coated with a double layer of titanium spray covered with HA (Figure 1);
2. monoblock long stems 120 mm long fully coated.

In this study, all the cementless tibial plate were regular stems (40 mm long). The design of the tibial plate and the stem is strictly the same in the cemented and cementless versions. The only difference between cemented and cementless implants is the state of the surface of the implant: fully coated with a double layer of titanium spray covered with HA in cementless tibial plate or roughness < 0.6 μ in the cemented version.

The mobile polyethylene insert (PI) is made of standard ultra high molecular polyethylene weight (UHMPEW), sterilized by ethylene oxide. Its design is specific to allow a total tibio-femoral congruence in the frontal and sagittal plane from full extension to full flexion. It can rotate over the highly polished tibia tray thanks to a 29 mm long peg freely rotating inside the tibia tray. The stabilizing device is 15.5 mm high and articulates to the inter-condylar femoral cage (Figure 2).

**Figure 3.**
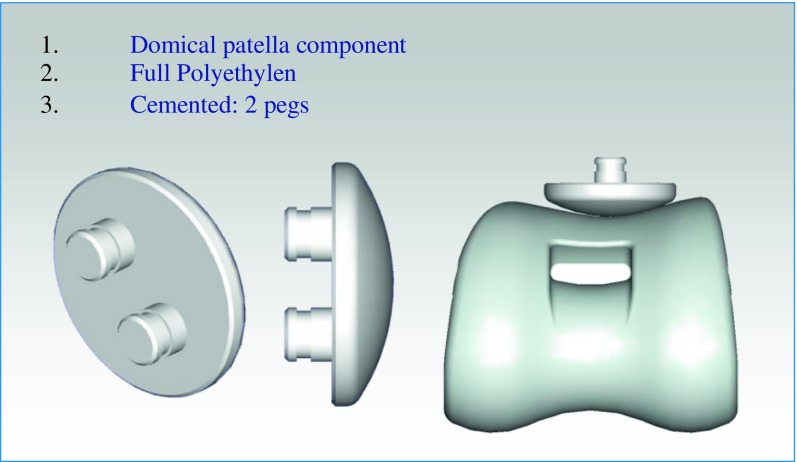
New Wave TKA^TM^ patellofemoral joint.

The patella component is a full polyethylene (PE) domical shape implant, cemented, 8 mm width with two pegs to ensure bone fixation (Figure 3).

### Surgical technique

The surgical approach is dependent on the type of deformity and ligament contracture. Usually an anteromedial approach is used in varus deformity, while an anterolateral approach in valgus deformity. Anterior tibial tubercle (ATT) osteotomy may be helpful in a stiff knee and/or patella infera to prevent patellar tendon avulsion. Pre-operative patella height assessment is calculated according to a new patella height index as reported by Caton et al. [1, 7] and Prudhon et al. [8]

The ancillary system refers to the intramedullary axis. In our technique, bone cuts are done independently but they can be related to each other. Pre-op planning on full leg length X-ray indicates how much valgus angulation has to be set up on the femoral cutting guide. The posterior cut of the femoral condyles refers to the anterior cortex of the femur. The tibial cut is perpendicular to the diaphysis axis of the tibia in frontal and sagittal plane. The design of the guide allows an extra-medullary check to secure the cut.

The ligament balance is usually done with trial implants.

Care has to be taken with the patella replacement to ensure the right positioning of the implant with respect to the functional centre of the patella.

Implants are cemented with a standard viscosity cement antibiotic loaded (AMINOFIX 1^TM^, Groupe Lépine, Genay, France). In all the knee replacements we have performed with the NEW WAVE^TM^ implant, whatever the fixation mode, we have replaced the patella with a full PE patellar component cemented with standard viscosity cement antibiotic loaded (AMINOFIX 1^TM^, Groupe Lépine, Genay, France).

Tourniquet was used in all the cases and was released just before cementation for the cemented implants or before implantation for the cementless components.

Full weight bearing and flexion are recommended immediately whatever the implant fixation. Patients are usually discharged in this series at day six and are followed with a clinical and radiographical examination at three months, six months, one year and every two years.

### Data collection

Data are collected on a computerized database (FileMaker Pro). Patient’s characteristics such as age at surgery, Charnley classification [9], aetiology, BMI and status (normal, overweight, obese, morbid obesity) functional evaluation, range of motion, surgical details, implant characteristics, pre, post op X-ray analysis, complications and functional outcomes are recorded.

**Figure 4.**
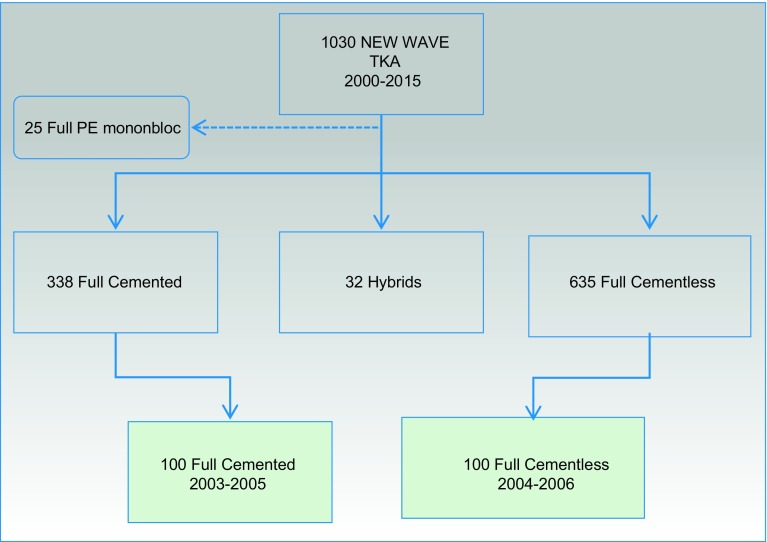
Flow chart of the cases selection.

Among the 1030 New Wave TKA^TM^ implanted from 2002 to 2015 by the single senior surgeon, we have identified 100 cemented New Wave TKA^TM^ implanted from 2003 to 2005 and 100 cementless New Wave TKA^TM^ implanted from 2004 to 2006. All these cases were primary replacement. We have excluded TKA after high tibia osteotomy (HTO), revision cases (uni or total knee revisions) or in patients born before 1921 (Figure 4).

Both populations have been statistically compared in terms of age, gender, aetiology and body weight status in order to obtain the most significantly compared analysis.

### Statistical analysis

Qualitative variables were presented as percentage, quantitative variables as mean and range. These variables were compared between both groups by chi-square or Fisher’s exact test. Quantitative variables were compared between both groups by Mann-Whitney test. The survival probability was assessed through the Kaplan-Meier method with 95% confidence interval. Differences in the survival probability were determined using log-rank test. Statistical analysis was performed with Stata software.

## Results

### Cemented series (Table 1)

Among 338 cemented New Wave TKA^TM^, 100 cases on 94 patients (59 females) have been included. The mean age at surgery is 73.16 years (44–83). Ten patients (10.6%) died of causes unrelated to knee surgery, six (6.4%) were definitely lost to follow up. We consider as lost to follow up, patients we were not able to follow at the regular outpatient visit, or patients we were not able to contact by phone or post mail. The mean follow-up (average time, in years, between surgery and latest revision) is 13.66 (14–12). Primary OA was the most frequent aetiology, 81% of medial OA in varus knee and 9% lateral OA in valgus deformity. Fifteen patients had previous surgery concerning meniscus, patella and ligament. According to Charnley classification, 17 had only one joint involved. One third had a normal weight and one third were obese. Anteromedial approach was used in 85 cases, in one case we had to do an ATT osteotomy. The mean gain of the Knee Society score was 57.2 for knee score and 41.6 for knee function. The post-operative range of motion is correlated to the pre-operative range of motion (Table 2). One patient developed a late infection at four years post-operative and underwent a global revision.

**Table 1.** Results of cemented and cementless series.

	Cemented	Cementless	*p* value
Number of cases	100	100	NS
Patients	94	95	
Females	59	57	
Age			NS
Mean age at surgery	73.16	72.25	
Mean follow-up	13.66	12.1	
Previous surgery			NS
None	85	79	
Meniscus surgery	9	9	
Ligaments reconstruction	2	5	
Distal patella realignment	2	4	
Articular fracture	2	3	
Patient status			NS
Normal	29	30	
Overweight	40	39	
Obesity	23	23	
Morbid obesity	8	8	
Charnley classification			NS
A	17	39	
B	74	56	
C	9	5	
Aetiology			NS
Medial OA grade 2	8	6	
Medial OA grade 3	63	59	
Medial OA grade 4	10	10	
Lateral OA grade 2	0	0	
Lateral OA grade 3	9	11	
Lateral OA grade 4	0	1	
Posttraumatic OA	3	3	
Rheumatoid Arthritis	4	5	
Patellofemoral OA	3	5	
Surgical approach			
ATT osteotomy	1	0	
Anterolateral	14	11	
Anteromedial	85	89	
HKA angle pre-op			NS
< 160	2	0	
160/164	6	17	
165/169	28	27	
170/173	35	19	
174/176	8	12	
177/179	4	1	
180	6	6	
181/183	1	2	
184/188	6	2	
189/192	3	4	
> 193	1	6	
Pre-operative range of motion			NS
> 137	34	34	
129/136	14	11	
121/128	12	8	
113/120	15	10	
105/112	8	11	
97/104	1	5	
< 97	1	12	
NR	16	9	
Flexum deformity pre-op			NS
> 15	4	11	
11–15	13	20	
5–10	28	25	
< 5	47	44	
NR	7	0

**Table 2.** Correlation of pre- and post-operative range of motion in cemented and cementless series.

	Cemented	Cemented	Cementless	Cementless
	Pre-op	Post-op	Pre-op	Post-op
Range of motion				
> 137	34	34	34	25
129/136	14	11	11	15
121/128	12	8	8	9
113/120	15	10	10	23
105/112	8	11	11	7
97/104	1	5	5	1
< 97	1	12	12	0
NR	16	9	9	20
Flexum deformity pre-op				
> 15	4	11	11	5
11–15	13	20	20	2
5–10	28	25	25	1
< 5	47	44	44	92
NR	7	0		
IKS score				
IKS knee	36.8	94	37.66	92.76
IKS function	50.6	92.2	44.2	95

Six patients were revised for loosening of one or both components. The mean interval between index surgery and revision was 5.4 years (1–13 years). (Table 3) Details are as follows:

- three cases (3%) in three patients with isolated aseptic loosening of the tibia component;
- three cases (3%) in three patients with bipolar (femur and tibia) aseptic loosening;
- no isolated aseptic loosening of the femoral component.

**Table 3.** Complications.

	Cemented	Cementless
ROM < 90° at three months (treated by manipulation under anesthesia)	9	6
Clunk (treated by arthroscopic synovectomy)	1	1
Stiffness (treated by arthroscopic arthrolysis)	1	3
Infection (treated by global revision)	1	0
Loosening (treated by revision)	6	2
Stiffness (treated by global revision)	1	0
Pain (treated by global revision)	1	0
Periprosthetic fracture (treated by global revision)	1	3
Periprosthetic fracture (treated by internal fixation)	0	2

Three other revisions were performed for:

- one case of unexplained pain;
- one case of stiffness;
- one case of periprosthetic fracture.

### Cementless series (Table 1)

Among 635 cementless New Wave TKA^TM^, 100 operations in 95 patients (57 females) have been included. Five patients (5.3%) died of causes unrelated to knee surgery, seven (7.4%) were lost to follow up. The mean age at surgery was 72.25 years (51–83). The mean follow-up was 12.1 years (11–13). Primary OA was the most frequent pathology 75% of medial OA in varus knee and 12% lateral OA in valgus deformity. Twenty-one patients had previous surgery concerning meniscus, patella and ligament. According to Charnley classification, 39 patients had only one joint involved. One third had a normal weight and one third were obese. Anteromedial approach was used in 89 cases, lateral approach in 11 cases. The mean gain of Knee Society Score was 55.1 for knee score and 50.8 for knee function (Table 2). The range of motion is correlated to the pre-operative range of motion (Table 2). No infections occurred in this series.

Two cases (2%) in two patients were revised for aseptic loosening of the tibial component at one and two years after index surgery (Table 3).

Four patients underwent a minor revision: one arthroscopic synovectomy for a clunck syndrome, three arthroscopic lysis for stiffness.

Five patients suffered a periprosthetic fracture (four femur, one tibia). Open fixation with a locking plate was performed in two cases, a global revision in three cases.

### Survival curve (Figure 5)

With the endpoint being revision of one or both components, survival probabilities at 11 years of follow-up were:

- cemented group: 90.2% CI95% [81.9–94.8] (78 arthroplasties still included in the analysis);
- cementless group: 95.4% CI95% [88.1–98.2] (69 arthroplasties still included in the analysis).

**Figure 5.**
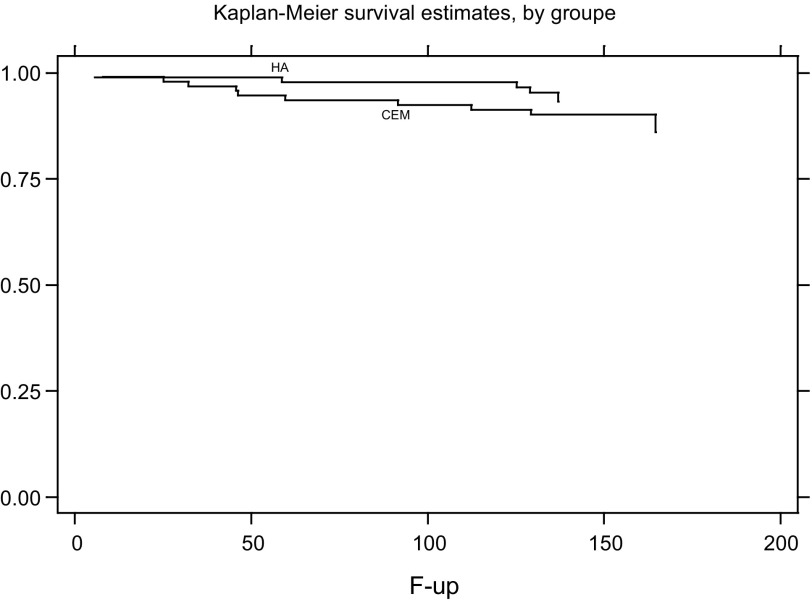
Survival curve.

The difference between each group was not significant (*p* = 0.32).

## Discussion

With a survival probability of 90.2% (cemented version) and 95.4% (cementless version), this total knee prosthesis performs as intended in primary total knee arthroplasty. We have observed more loosening in the cemented series than in cementless (six cases vs. two cases). Actually due to the small size of the sample the difference is not statistically significant. However, our feeling is that we have observed less fixation failure with the cementless component than with the cemented one. On the other hand, we have observed more periprosthetic fractures with the cementless TKA but differences are not significant. This is one of the weaknesses of this study which is a retrospective nonrandomized cohort. No statistical differences could be found between cemented and cementless implants. The strengths of the study are length of follow-up and comparison of fixation mode between two series which are similar from an epidemiologic point of view.

### Cementless fixation

Historically TKA components were fixed to the bone thanks to a cementing technique as used in the Total Hip Arthroplasty (THA). Satisfying mid-term results with cementless fixation in THA, in the early 1980s, had led surgeons to introduce this fixation mode [3] (PCA group – Hungerford, Kenna, Krackow).

Cementless fixation was obtained by a macroporous coating with two layers of metallic beads. To ensure immediate stability of the implant, the authors also introduced an original ancillary device to get the closest contact between the implant and the host bone. Early results were excellent but failures occurred mainly due to the beads’ release and migration in the prosthetic joint [3–5]. Miller and Gallante [10] addressed the metallic failures of the PCA TKR by using a titanium fibre-mesh coating (Zimmer^TM^). It has been suggested that the poor design of these early components could be responsible for these bad outcomes. Previous failures have led the surgeons to shift to a cemented fixation as demonstrated in the National Joint Registry data [11, 12]. Interest in cementless fixation in TKA has increased in many parts of the world and especially in France.

The theoretical advantages of cementless TKA are bone stock preservation, cement debris protection and the potential to achieve biologic fixation of the implant to the bone. Cementless implants rely on a porous or roughened surface to facilitate bone formation. The initial stability obtained at surgery influences long-term fixation [13], which is important to prevent micromotion compromising the chance of achieving osseointegration.

### Added value of a double-layer titanium and hydroxyapatite (HA) coating

Hydroxyapatite (HA) is a bioactive coating added to the metal of a cementless TKA. The new wave cementless implant is made of cobalt chromium alloy. It can be used with or without cement. In the cementless version, the implant is totally coated under vacuum with a double layer of titanium spray (120 μm) covered with HA (80 μm). HA has been shown to accelerate bone integration and so to decrease micromotion of the tibia component and to increase fixation of both components [14–16]. Cross and Parish [14] reported a series of 1000 patients with HA-coated cementless TKA at nine years’ follow-up with a 0.5% revision rate for aseptic loosening. Epinette and Manley [6] found a survivorship of 98.14% at a mean follow-up of 11.2 years (endpoint is mechanical failure) in 146 primary TKA treated with an HA-coated cementless TKA. Voigt and Mosier [17] in a meta-analysis of 926 arthroplasties conclude that HA-coated implants may provide better durability than other forms of fixation including cemented TKA.

With a 95.4% CI95% [88.1–98.2] (endpoint: revision of one or both components), our results are consistent with results previously reported.

### New technologies in cementless fixation

Trabecular metal is a biomaterial made of Tantalum with porosity and mechanical properties resembling trabecular bone [18]. Other new concepts are introduced in cementless fixation: BIOFOAM (MicroPort Orthopedics Inc) is one of the several titanium foams created by various manufacturers. Additive manufacturing using electron beam melting (EBM) is also coming to orthopaedic implants.

### Rotating tibial plate

Rotating tibial components were introduced 20 years ago in order to decrease the stresses at the interface between implant and bone. Buechel et al. [19] reported in 2001 a series of 140 New Jersey LCS TKR at 16 years’ follow-up with a survivorship of 100%. One of the main complications of this kind of device was dislocation or the so-called spinout of the rotating PE insert. To prevent this particular complication, a New Wave polyethylene insert is characterized by a specific design to allow a total tibio-femoral congruence in the frontal and sagittal plane from full extension to full flexion. It can rotate over the highly polished tibia tray thanks to a 29 mm long peg freely rotating inside the tibia tray. The stabilizing device is 15.5 mm high and articulates to the inter-condylar femoral cage (Figure 3). In our series we did not observe such a complication.

### Cemented versus cementless fixation

In our series, even though the survival probability seems to be better in the cementless group, no statistical differences could be found between cemented and cementless New Wave TKA^TM^. These results are consistent with those of randomized controlled trials [21, 22] or series with long-term follow-up [23] comparing cemented and cementless fixation (Table 4) and Gandhi et al. [24] in his meta-analysis or, Arnold et al., in his systematic literature review analysis [25].

**Table 4.** Randomized controlled trials comparing cemented and cementless fixation (survivorship endpoint = revision for all causes).

Author, year	Study type	*N* prosthesis	Follow-up (year)	Cemented (%)	Hybrid (%)	Cementless (%)	Cementless + HA (%)	*p*
Kim et al. [20], 2014	RCT	160	17	100		100		NS
Beaupré et al. [21], 2007	RCT	70	5	97.5			97.5	NS
Baker et al. [22], 2007	RCT	269	10	91.7		93.3		NS
Parker et al. [23], 2001	RCT	100	14		65.0	50.0		NS

According to our results we can conclude that cementless fixation has demonstrated its reliability. Mid- and long-term results are similar to cemented fixation which is still the most common method used throughout the world. This fact seems paradoxal when comparing the fixation mode between hip and knee replacement.

## Conclusion

With a survival probability of 90.2% (cemented version) and 95.4% (cementless version), New Wave TKA^TM^ total knee prosthesis performs as intended in primary total knee arthroplasty. No statistical differences could be found between cemented and cementless implants.
